# Inhibition of planktonic growth and biofilm formation of *Staphylococcus aureus* by entrectinib through disrupting the cell membrane

**DOI:** 10.3389/fmicb.2022.1106319

**Published:** 2023-01-09

**Authors:** Shanghong Liu, Yanpeng Xiong, Haitao Xiao, Jinxin Zheng, Zewen Wen, Duoyun Li, Qiwen Deng, Zhijian Yu

**Affiliations:** ^1^School of Pharmaceutical Sciences, Health Sciences Center, Shenzhen University, Shenzhen, China; ^2^Department of Infectious Diseases and the Key Lab of Endogenous Infection, Shenzhen Nanshan People’s Hospital, The Sixth Affiliated Hospital of Shenzhen University Health Science Center, Shenzhen, China

**Keywords:** entrectinib, biofilm, *Staphylococcus aureus*, proteomic, antibacterial

## Abstract

Over the last few decades, *Staphylococcus aureus* infection remain a major medical challenge and health concern worldwide. Biofilm formation and antibiotic resistance caused by *S. aureus* make it difficult to be eradicated from bacterial infections in clinics. In this study, our data demonstrated the antibacterial and excellent anti-biofilm activity of entrectinib against *S. aureus*. Entrectinib also exhibited the good safety, suggesting no toxicity with antibacterial concentration of entrectinib toward the erythrocytes and mammalian 239 T cells. Moreover, entrectinib significantly reduced the bacterial burden of septic tissue in a murine model of MRSA infection. Global proteomic analysis of *S. aureus* treated with entrectinib showed significant changes in the expression levels of ribosomal structure-related (rpmC, rpmD, rplX, and rpsT) and oxidative stress-related proteins (Thioredoxin system), suggesting the possible inhibition of bacterial protein biosynthesis with entrectinib exposure. The increased production of reactive oxygen species (ROS) was demonstrated in the entrectinib-treated *S. aureus*, supported the impact of entrectinib on the expression changes of ROS-correlated proteins involved in oxidative stress. Furthermore, entrectinib-induced resistant *S. aureus* clone was selected by *in vitro* induction under entrectinib exposure and 3 amino acid mutations in the entrectinib-induced resistant *S. aureus* strain, 2 of which were located in the gene encoding Type II NADH: quinoneoxidoreductase and one were found in GTP pyrophosphokinase family protein. Finally, the bactericidal action of entrectinib on *S. aureus* were confirmed by disrupting the bacterial cell membrane. Conclusively, entrectinib exhibit the antibacterial and anti-biofilm activity by destroying cell membrane against *S. aureus*.

## Introduction

*Staphylococcus aureus* is a major human and animal pathogen that prominently causes a wide range of hospital-and community-acquired infections in the clinic (Yang et al., 2017; Thwaites et al., 2018). The spectrum of infectious diseases caused by *S. aureus* range from minor skin and soft tissue infections, endocarditis, osteomyelitis, and pneumonia to life-threatening infections such as septic shock or critical pneumonia (Maurer et al., 2015; Tong et al., 2015). In recent years, the emergence of multiple drug-resistant *S. aureus*, including methicillin-resistant *S. aureus*, vancomycin-resistant *S. aureus*, and linezolid-resistant *S. aureus*, has aroused great concern among the public (D’Costa et al., 2011; Shariati et al., 2020; Sun et al., 2020). The rapid transmission and the development of antibiotic resistance in *S. aureus* has widely limited the choices of antimicrobial treatment (Chambers and Deleo, 2009). Recently, the gradual increase of the treatment failure caused by *S. aureus* infection has been increasingly reported in antimicrobial treatment with the last-resorted antibiotics, such as vancomycin, linezolid and daptomycin (Katayama et al., 2017; Zhou et al., 2018; Sun et al., 2020). Therefore, in order to combat the difficult-to-treat infection caused by *S. aureus*, the development of novel antibacterial agents is urgently needed.

The biomaterials or the medical devices colonized with *S. aureus* are prone to form biofilm and it has become a significant challenge to improve the treatment outcome of biofilm-related implanted infection in clinics (Heim et al., 2020). The ingredients of biofilm mainly involves extracellular polymeric substances (EPS), which are composed of extracellular polysaccharides, proteins, and extracellular DNA (eDNA). The biofilm cells are closely adhesive to each other and the biofilm structure is morphologically similar to a barrier, which can limit the diffusion and penetration of antimicrobial drugs (Yu et al., 2015; Flemming et al., 2016; Schramm et al., 2020). In addition, the high frequency of *S. aureus* clinical isolates displays the capacity to form biofilm, which often greatly reduces their sensitivity to antibiotics and protects themselves from immune clearance and immunotherapy. Biofilm formation has become one of the most important factors that caused the failure of antimicrobial treatment against *S. aureus* infection (Periasamy et al., 2012; Lister and Horswill, 2014; Shi et al., 2021). Therefore, the antimicrobial agents coupling with the anti-biofilm activity would greatly improve the clinical outcome of antimicrobial treatment against the biofilm-related or intractable *S. aureus* infection.

Drug repurposing is often explained as a strategy for reusing existing or approved drugs in unrelated other diseases. Previous reports support drug repurposing, as one of the excellent pathways for the development of the novel antimicrobial agents (Cruz-Muniz et al., 2017). Our previous reports have indicated the discovery of antimicrobial agents with anti-biofilm activity from the drugs that have previously been approved for non-antimicrobial uses, supporting Drug repurposing as an successful and economical approach for the discovery of the novel antimicrobial agents (Zheng et al., 2021; Shang et al., 2022; Wen et al., 2022). Moreover, entrectinib is firstly screened as a potential antimicrobial agent by us from the FDA-approved drug library. Entrectinib, an oral, selective tyrosine kinase inhibitor, has been approved for the treatment of solid tumors harboring NTRK, ROS1, and ALK fusions several years ago. Entrectinib is considered as a weak p-glycoprotein substrate that can penetrate the brain–blood barrier. The effective concentrations within the central nervous system after oral administration of entrectinib facilitate it to exhibit the potent systemic antitumor activity in this organ (Dziadziuszko et al., 2021). Some reports showed excellent safety in animal experiments with doses up to 240 mg/kg/d for 14 consecutive days (Fischer et al., 2020; Frampton, 2021). The mounting clinical studies further showed its excellent safety in humans, whereas its antibacterial activity has never been reported.

Hence, the purpose of this study was to evaluate the antibacterial and anti-biofilm activity of entrectinib against *S. aureus*. In addition, the effect of entrectinib on bacterial protein expression was investigated by label-free global proteomics, and the potential antibacterial mechanism of entrectinib was performed by *in vitro* induction of entrectinib resistant *S. aureus* and whole gene sequencing. Furthermore, disruption of the cell membrane of *S. aureus* by entrectinib was investigated as a possible antibacterial mechanism.

## Materials and methods

### Bacterial strains and growth conditions

*Staphylococcus aureus* and *E. faecalis* clinical isolates employed in this study were retrospectively collected from the 6th Affiliated Hospital of Shenzhen University Health Science Center and identification of these bacteria isolates was performed by MALDI-TOF mass spectrometry (IVD MALDI Biotyper, Bruker, Karlsruhe, Germany), as in our previous reports. *Staphylococcus aureus* SA113and USA300 strains were purchased from the American type culture collection (ATCC). Strains were grown in tryptic soy broth (TSB) at 37°C with shaking at 200 rpm unless otherwise stated.

### Determination of the minimum inhibitory concentration

To determine the minimum inhibitory concentration (MIC) of entrectinib, all the strains (1:1,000) were grown in Cation-Adjusted Mueller Hinton II Broth (CAMHB) on a 96-well round-bottomed microplate and exposed to a series of half dilutions of entrectinib (1.56, 3.13, 6.25, 12.5, 25, 50, 100, and 200 μM). CAMHB with equal volumes of entrectinib in Dimethyl sulfoxide (DMSO) was used as a control. The MIC readings were performed after incubation at 37°C for 24 h and were defined as the lowest concentration of visually inhibiting bacteria growth.

### Growth curve assay

All the strains (1:200 dilution) were grown in TSB and exposed to different concentrations (6.25, 12.5, and 25 μM) of entrectinib at 37°C shaking at 200 rpm. Bacterial growth curves in TSB without entrectinib in the plate were used as a control. The growth curves were measured by optical density at 600 nm (OD_600_) at 1 h time intervals for 24 h under the Bioscreen C (Turku, Finland). The experiments were repeated at least three times in triplicate.

### Biofilm inhibition assay

The capacity for entrectinib to inhibit the biofilm was assessed according to previous reports (Zheng et al., 2022). The different concentrations of entrectinib were inoculated with the *S. aureus* in the 96-well polystyrene plates with TSBG (TSB with 0.5% glucose) containing various concentrations of entrectinib (0, 6.25, and 12.5 μM) at 37°C for 24 h without shaking. Then stained with crystal violet solution and determined using a spectrophotometer at a wavelength of 570 nm (OD_570_). The experiments were performed in triplicate at least three times. The confocal images were acquired using a Confocal Laser Scanning Microscope (FV3000, OLYMPUS, Japan) with a 100× oil immersion objective, and the bacteria cells in the biofilm were stained by LIVE/DEAD (1 μM SYTO9 and 1 μM propidium iodide; Thermo Fisher Scientific, Houston, TX).

### Time-kill assay

*Staphylococcus aureus* YUSA145 and CHS101 were cultured for 24 h in TSB, then 1:200 diluted in TSB, after growing to OD_600_ reaching 0.5 at 37°C. The strains were exposed to the 25, 50, and 100μM entrectinib or without the drug, respectively, for 3, 6, and 24 h and resuspended with 0.9% NaCl. Vancomycin (8 μg/ml) was used as the positive control. The samples were diluted and plated on TSB agar plates, and the calculation of viable cells was identified by CFU counting. All the experiments were repeated in triplicate.

### Selection and whole-genome sequencing of entrectinib-induced resistant *Staphylococcus aureus*

*Staphylococcus aureus* isolates CHS101 were induced under the *in vitro* pressure of entrectinib with the initial concentration from 12.5 μM with increasing induction concentration every 5 days for 60 consecutive days until to 200 μM. Three individual derivative clones were picked and isolated at day 60 for subsequent three consecutive generations without entrectinib treatment in TSA plates. During the induction, *S. aureus* clone which could survive in the 200 μM entrectinib was picked and defined as entrectinib tolerant derivatives. The entrectinib tolerant derivatives with significant MIC elevation of entrectinib was determined as the entrectinib-induced resistant *S. aureus*. The total DNA was extracted from entrectinib-induced resistant *S. aureus* clone using the MiniBEST Bacteria Genomic DNA Extraction Kit Ver.3.0 (Takara Biotechnology, Dalian, China). Using NEBNext Ultra DNA Library Prep Kit for Illumina (NEB, United States) to generate the sequencing libraries. The whole genome was sequenced by Illumina HiScanSQ using PE150 chemistry (Illumina) and analyzed by BWA-MEM software (v0.7.5a) 2 with standard parameters. The whole genome sequencing of entrectinib-induced resistant *S. aureus* was uploaded to the NCBI database with the biosample accession SAMN31720757. Compared the sequence of parenteral isolate CHS101 with the bioproject accession PRJNA889679and the entrectinib-induced resistant *S. aureus* using the MUMmer comparison software. Reliable SNPs were obtained after using BLAST, TRF, and Repeatmasker software to predict the repeat region of the sequencing and filter out the SNPs.

### Proteomic analysis

*Staphylococcus aureus*SA113 was grown in TSB with entrectinib (12.5 μM) or DMSO for 2 h at 37°C. To harvest the bacteria, the cultures were centrifuged at 5000 rpm for 10 min at 4°C and transferred to a new 2 ml screw-cap tube. The RIPA buffer (Beyotime, Shanghai, China) and glass beads were added, followed lysed by a cell disruption device at 70 Hz for 3 min. The proteins were reduced with 10 mM DTT (Sigma-Aldrich Co., St. Louis, MO) for 1 h at 70°C, then the alkylation was carried out using 50 mM iodoacetamide (IAA, Sigma-Aldrich) for 15 min at room temperature in the dark. The samples were desalted, and the buffer was changed to 0.5 M ammonium bicarbonate buffer. Then the proteins were digested with trypsin (Promega, Madison, WI). Peptides were separated on a C18 tip column (75 μm × 250 mm, Acclaim PepMap RSLC, 2 μm) and analyzed with a Q Exactive Plus mass spectrometer (Thermo Fisher Scientific Inc.). The data were compared with the Uniprot reference proteome of *S. aureus* (strain NCTC 8325/PS 47) reference proteome database (UP000008816.fasta; 2,889 entries; downloaded in 20.02.2021).

### Cytotoxicity assay

Assays for cellular viability were conducted using the cell counting kit-8 (CCK-8). The 293 T cells were cultured in T75 EasY Flask using Dulbecco’s modified eagle medium (DMEM) at 37°C in the presence of 5% CO_2_. An aliquot of 100 μl of the cell suspension (containing 1 × 10^4^ cells) was added into a 96-well plate and incubate at 37°C for 24 h. After the old medium was removed, fresh DMEM containing different concentrations of entrectinib was added to the corresponding wells and incubated for another 24 h. An aliquot of 10 μl CCK-8 reaction solution was added to each well of the plate, and the plate was incubated at 37°C for 1 h, and then the optical density was detected at 450 nm.

### Mouse wound infection model

This infection model was conducted with slight modifications based on previously reported methods (Cho et al., 2011; Xie et al., 2021; Gordon et al., 2022). An intraperitoneal injection of sodium pentobarbital at a 50 mg/kg dose was used to anesthetize mice before surgery to construct wound models caused by MRSA. The 6-mm diameter full-thickness wounds were pierced in both sides of the back using a biopsy punch after the back hair was shaved and the skin was rinsed with 75% alcohol. Then, 10 μl *S. aureus* USA300 suspension (1 × 10^6^ CFU/ml) was added to the wound, and the wound site was covered with a Tegaderm dressing to prevent contamination. Within 24 h of infection, mice were randomized, and then 10 μl of different treatments were applied: 0.9% saline, vancomycin (2 mg/ml), and entrectinib (2 mg/ml), three treatments were administered every 8 h. The mice were sacrificed 4 h after the last dose, and the wound site was excised, weighed, and homogenized in PBS solution. The homogenized suspension was serially 10-fold diluted with saline, and 100 μl amounts of appropriate dilutions were inoculated onto tryptone soy agar (TSA) plates in triplicate. The numbers of *S. aureus* clones were counted and bacterial burdens in wound homogenates (CFU/g) were calculated after 24 h of incubation at 37°C.

### Molecular docking

The molecular structure pdb file for *S. aureus* NDH-2 protein (5NA4) was downloaded from the Protein Data Bank (PDB). Using the Protein Preparation Wizard module of Schrödinger software to hydrogenate, repair missing residues, and optimize the structure, and water molecules. The molecular 3D structure file for entrectinib was downloaded from PubChem. The binding model of NDH-2 protein and entrectinib was predicted by molecular docking. Briefly, the best binding pocket of NDH-2 protein was chosen by structure-based cavity detection, then the docking based on AutoDockVina was performed to investigate the best binding site of entrectinib according to the Vina score (kcal/mol) in the pocket (Liu et al., 2022).

### Cytoplasmic membrane depolarization assay

The membrane depolarization assessment was performed with slight modifications from a previously reported method (Wen et al., 2022). Overnight culture *S. aureus* SA113 was diluted in 4 ml PBS to 1× 10^6^CFU/mL, probe DiBAC_4_(3) (1 μM) was added, and the suspension was incubated for 30 min, followed by the addition of KCl to a final concentration of 0.1 M to balance the cytoplasmic and external K^+^ concentration. An aliquot of 90μl bacteria suspension was placed in a 96-well black plate and then fluorescence intensity was recorded on a microplate reader (excitation λ = 492 nm, emission λ = 518 nm) until it remained stable, then 10 μl PBS containing entrectinib was added to final concentrations of 1 × and2 × MIC, 0.1% DMSO was used as the negative control, and then the fluorescence intensity was continuously detected 5 min intervals for 50 min.

### ROS assessment

Overnight culture *S. aureus*SA113 was diluted in 2 ml PBS to1 × 10^7^ CFU/ml, probe DCFH-DA (5 μM) was added, the suspension was incubated for 30 min in dark, then the bacterial cells were washed with PBS twice to remove the DCFH-DA outside the cell and then diluted in PBS to 1 × 10^7^ CFU/ml. An aliquot of 90 μl the bacterial suspension was placed in a 96-well plate, followed by 10 μl of PBS (with entrectinib at 2 × and 4× MIC), and the DMSO was added as a negative control. The fluorescence intensity was recorded continuously on a microplate reader for 1 h (excitation λ = 492 nm, emission λ = 518 nm).

### Transmission electron microscopy assay

To check the effect of entrectinib on bacterial cell membrane damage, Transmission electron microscopy (TEM) was performed. *S. aureus* SA113 (1:200 dilution) was inoculated with TSB at 37°C shaking at 200 rpm for 3 h. The bacteria samples were centrifuged at 5,000 rpm for 10 min at 4°C to harvest bacteria, the pellets were collected, washed twice with PBS, resuspended in PBS, and then divided into two aliquots. The aliquots were treated with 100 μM entrectinib or DMSO for 1 h. After the centrifuge, the bacteria precipitation was resuspended in the fixative. The samples were fixed with 1% OsO4 in 0.1 M PB (pH 7.4) and EMBed 812-embedded. Next, 60-80 nm sections were stained with 2% uranium acetate saturated alcohol solution and 2.6% Lead citrate using a standard protocol and then analyzed with TEM.

### Statistical analysis

Continuous variables were analyzed by Student’s t test and one-way factorial analysis of variance (ANOVA) using SPSS version 24.0 software (SPSS, Inc., Chicago, IL, United States).

### Data availability

The whole-genome sequencing files of the entrectinib-tolerant CHS101 clone were deposited in the NCBI database with the Biosample accession SAMN31720757 and the reference sequence the parenteral isolate CHS101 with the BioProject accession PRJNA889679. The raw data of whole-genome sequencing were posted in the Sequence Read Archive (SRA) database under BioProject accession number PRJNA901618 in NCBI. The raw proteomics data are deposited in the ProteomeXchange Consortium through the partner iProX system with dataset identifier PXD038342.

## Results

### Antibacterial activity of entrectinib against *Staphylococcus aureus*

**Figure 1:**
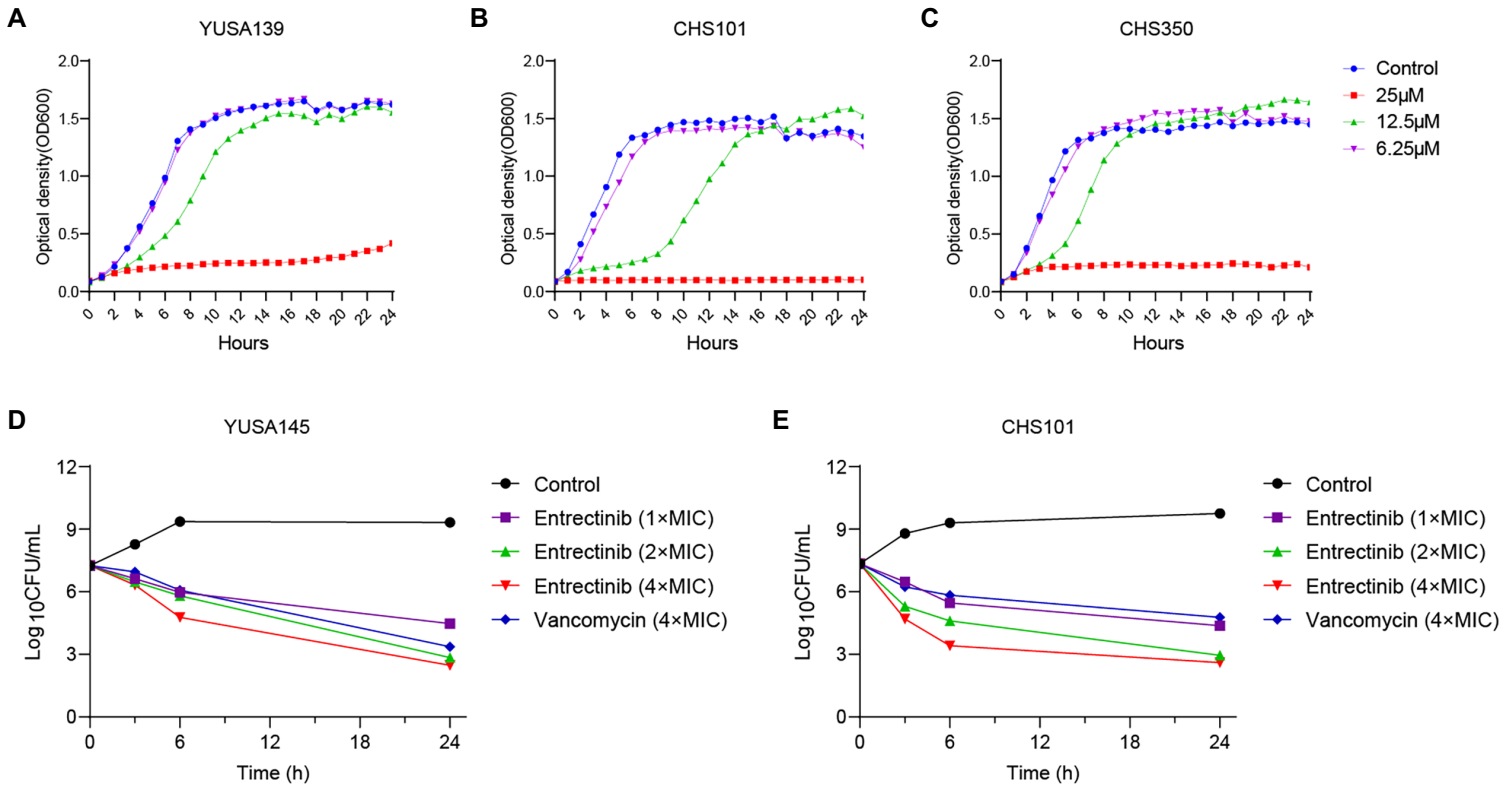
The inhibition and Time-killing assay of *Staphylococcus aureus* planktonic growth by entrectinib. Impact of entrectinib at different concentrations (1/4×, 1/2×, and 1× MIC) of entrectinib on the bacterial growth of MRSA YUSA139 **(A)**, MRSA CHS350 **(C)**, and MSSA CHS101 **(B)** planktonic cells under the Bioscreen C. **(D,E)** Time-killing assay of entrectinib with various concentrations (1×, 2×, and 4× MIC) against the planktonic growth of *S. aureus* isolates MRSA YUSA145 and MSSA CHS101.

Initially, MIC of entrectinib was investigated for evaluating its antibacterial activity against clinical isolates of gram-positive bacteria, suggesting its range from 12.5to 25 μM in *S. aureus* and *E. faecalis* (Table 1). Subsequently, inhibition of planktonic growth of *S. aureus* by entrectinib were measured in clinical isolates of *S. aureus* YUSA139, CHS350, and CHS101 at different sub-MIC concentrations of entrectinib (6.25, 12.5, and 25 μM) by automatic instruments, demonstrating the potently antibacterial activity of entrectinib at the concentration of 25 μM (Figure 1). Moreover, the *in vitro* bactericidal ability of entrectinib was investigated by time-kill kinetics assay with *S. aureus* strains YUSA145 (MRSA) and CHS101 (MSSA), showing the excellent bactericidal activity of entrectinib on the planktonic cells of *S. aureus* with a dose-dependent manner. Worthy of our attention, after 6 h of exposure to entrectinib with the concentration of 4× MIC, its bactericidal effect was even more potent than that with vancomycin concentration of 4× MIC (Figures 1D,E).

**Table 1:** The MIC distribution of entrectinib against *Staphylococcus aureus* and *E. faecalis*.

Organism	Number	MIC of entrectinib (μM)		MIC_50_/MIC_90_
		25	50	
MSSA	34	31 (91.2%)	3 (8.8%)	25/25
MRSA	16	15 (93.8%)	1 (6.2%)	25/25
*E. faecalis*	16	5 (31.3%)	11 (68.7%)	50/50

MRSA, methicillin-resistant *Staphylococcus aureus*; MSSA, methicillin-susceptible *Staphylococcus aureus*.

### Entrectinib significantly inhibits *Staphylococcus aureus* biofilm formation

**Figure 2:**
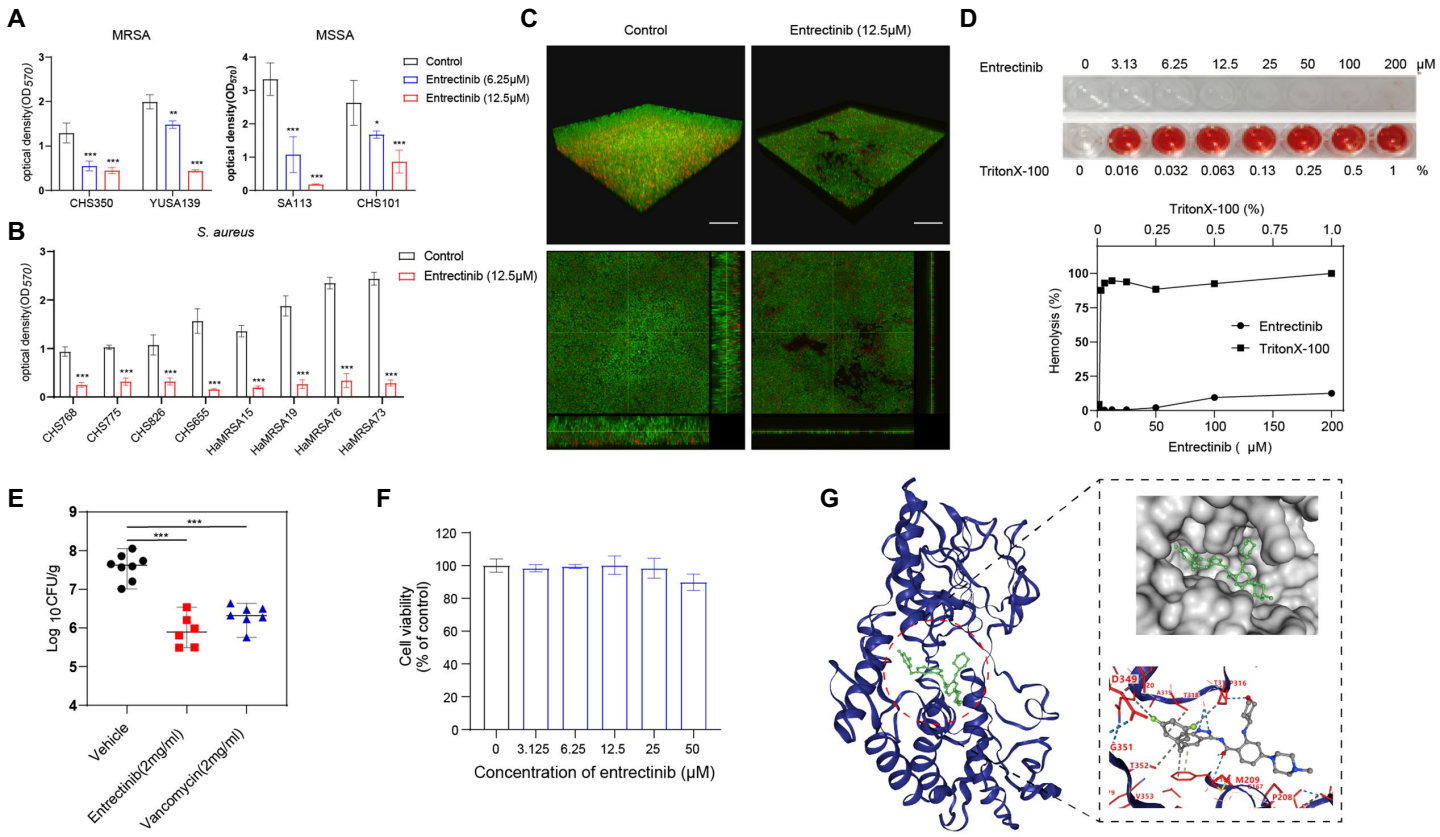
**(A)** Anti-biofilm activity of entrectinib at various concentrations (1/2× and 1/4× MIC) against *Staphylococcus aureus*. **(B)** The biofilm formation of 8 clinical isolates of *S. aureus* was significantly inhibited by 12.5 μM entrectinib using crystal violet staining. The data presented were the average of three independent experiments (mean ± SD). **(C)** Impact of entrectinib on *S. aureus* SA113 biofilm formation at 12.5 μM was investigated by CLSM. Two images below on the right and bottom sides clearly showed the thickness of the *S. aureus* biofilm, respectively. Scale bar 20 μm. **(D)** The hemolytic activity of entrectinib against the rabbit erythrocytes. The upper represents the observation of entrectinib’s hemolysis in 96-well cell plates and the down represents the comparison of the amount of its hemolytic activity quantified with OD450. **(E)** Comparison of the CFU counting assay of MRSA in the local septic tissue of the *S. aureus* wound murine infection model treated with saline, entrectinib, or vancomycin, respectively (*n* = 6 mice/group). **(F)** The cytotoxicity assay of entrectinib against 293 T cells using the CCK-8. **(G)** The molecular docking of NDH-2protein (blue) and entrectinib (green) suggested the direct interactions between NDH-2 protein and entrectinib. Using a ball-and-stick style, the entrectinib was shown with the elements in a variety of color combinations (blue for nitrogen atom; red for oxygen atom; green for fluorine atoms).MRSA: CHS350 and YUSA139; MSSA: SA113 and CHS101. Compared with control, *p < 0.05; **p < 0.01; ***p < 0.001; (Student’s *t*-test).

The impact of entrectinib at sub-MIC concentrations (1/4× and 1/2× MIC) on *S. aureus* biofilm formation was evaluated in 2 clinical isolates of MRSA and 2 clinical isolates of MSSA by crystal violet staining, indicating the significant inhibition of biofilm formation of *S. aureus* by entrectinib (6.25 and 12.5 μM; Figure 2A). In addition, the sturdy inhibition of biofilm formation by entrectinib at sub-MIC concentrations was confirmed in 8 *S. aureus* clinical isolates (Figure 2B). Furthermore, the influence of entrectinib on the biofilm formation of *S. aureus* was investigated by confocal laser scanning microscopy using fluorescence staining and our data indicated the biofilm formation in the control group was about 9 times thicker when compared with that in the entrectinib-treated group, showing the significant inhibitory effect of entrectinib on the biofilm formation of *S. aureus* (Figure 2C).

### Cytotoxicity assay and *in vivo* anti-infective effect of entrectinib

The toxicity of the entrectinib to mammalian cells was assessed by determining its hemolytic activity against the rabbit erythrocytes, showing no toxicity below 200 μM toward the rabbit erythrocytes (Figure 2D). The cytotoxicity of entrectinib on the 293 T cells was investigated using CCK-8, suggesting no impact of this chemical on the cell viability of 293 T cells with various concentrations of entrectinib after 24-h treatment (Figure 2F). This data indicated that entrectinib with effective concentrations that could inhibit bacterial growth and biofilm formation seemed no cytotoxicity to normal mammalian cells. Subsequently, the antibacterial activity of entrectinib in a mouse full-thickness wound infection model treated with entrectinib after 24 h were further evaluated. The bacterial burden of septic tissues in the entrectinib-treated group showed significantly low when compared with the control and the efficacy of antimicrobial treatment of entrectinib in this model was mostly equal to that of vancomycin (Figure 2E).

### Global proteomic response of *Staphylococcus aureus* treated with entrectinib

**Figure 3:**
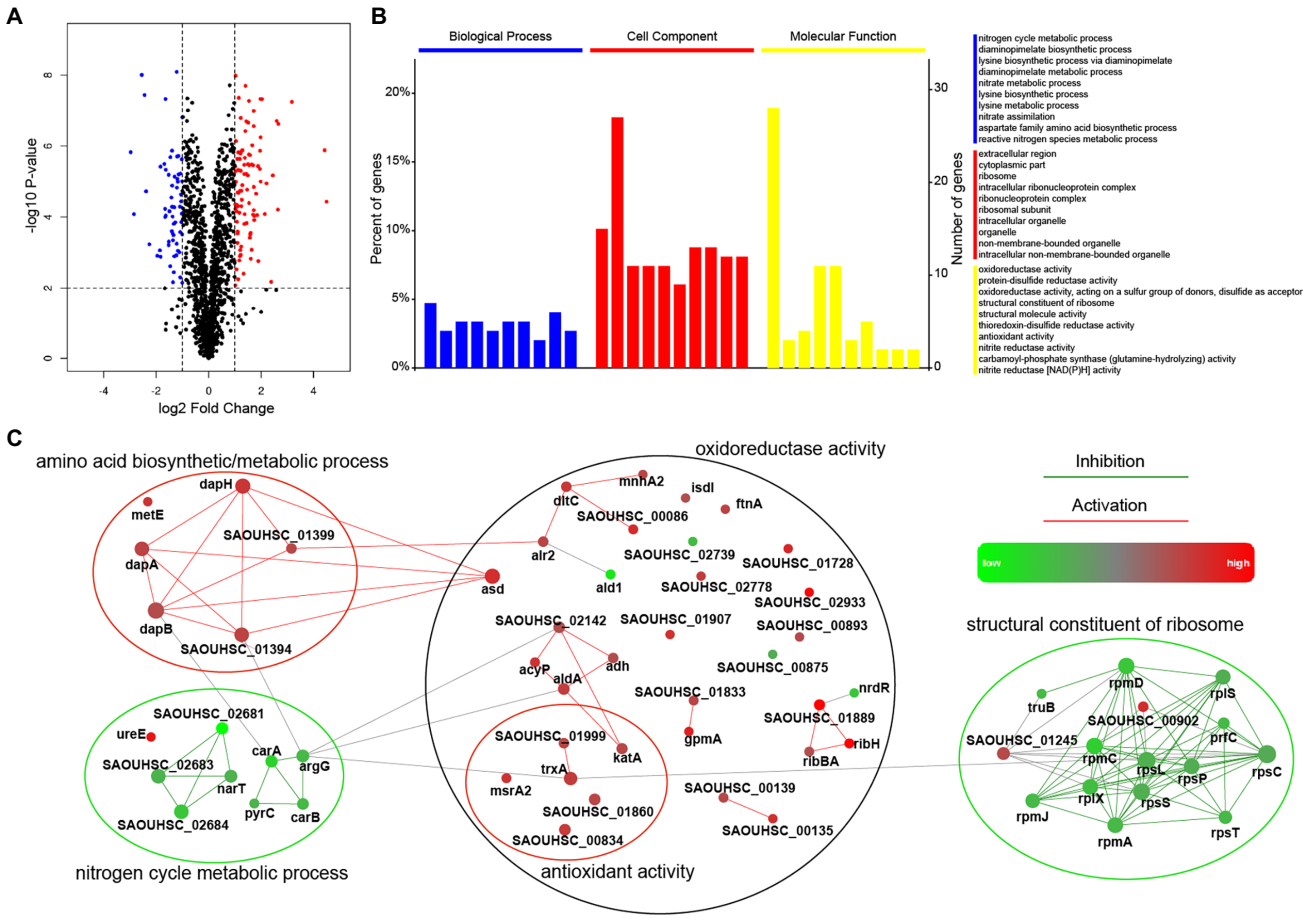
Comparison of the significantly differentially expressed proteins in *Staphylococcus aureus* treated with or without entrectinib. **(A)** The volcano plots of the proteomics of *S. aureus* SA113 after being treated with entrectinib (12.5 μM) compared to that in the untreated control. Blue dots represented the upregulated proteins and the red dots represented the downregulated proteins. **(B)** The GO enrichment of the significantly differentially expressed proteins between two groups. The blue, red, and yellow columns indicated the classified proteins that have function in the biological processes, cellular components, and molecular functions, respectively. **(C)** Protein–protein interaction network (PPI) of the differentially expressed proteins between two groups. Each dot represented a protein and the protein–protein lines represented their link and interaction.

The proteomic response of *S. aureus* strain SA113 treated with entrectinib concentration of 12.5 μM for 2 h was analyzed by a quantitative label-free proteomic analysis with the mass spectrometry. Totally, 1,640 proteins were identified confidently (matched peptides ≥ 1), and 173 differentially expressed proteins were determined (log_2_foldchange ≥ |1|, *p*-value ≤ 0.05), including 106 upregulated proteins and 67 downregulated proteins (Supplementary Table S1). The results showed that the significantly upregulated proteins were involved in the oxidoreductase activity, antioxidant activity, lysine biosynthetic/metabolic process, aspartate family amino acid biosynthetic process, and protein-disulfide reductase activity. Moreover, the significantly decreased expressed proteins were mainly correlated with the nitrate metabolic, nitrogen cycle metabolic process, structural constituent of ribosome, and structural molecule activity (Figure 3).

### Genetic mutations in entrectinib-induced resistant *Staphylococcus aureus*

The *in vitro* induction of parenteral *S. aureus* CHS101were performed under exposure to a series of entrectinib concentrations and the entrectinib-induced resistant *S. aureus* was selected after the *in vitro* induction for 60 days. Then, entrectinib-induced resistant *S. aureus* was identified with an eight-fold increase of MIC to 200 μM. Then, the genetic mutations between the entrectinib-induced resistant strain and the parenteralCHS101 strain were analyzed by performing the whole genome sequencing, suggesting adaptation mutation in 3 coding genes were found in the entrectinib-induced resistant *S. aureus* strain, including a premature stop codon and 2 non-synonymous mutations (Supplementary Table S2). Two genetic mutation points in Type II NADH: quinoneoxidoreductase of entrectinib-induced resistant *S. aureus* strain and another mutation in GTP pyrophosphokinase family protein were determined. Type-II NADH: quinoneoxidoreductases (NDH-2) is a membrane-binding protein involved in respiratory chains and the only enzyme with NADH: quinoneoxidoreductase activity expressed in *S. aureus*. Previous studies reported its crucial role in the *S. aureus* growth (Sena et al., 2018). Furthermore, the binding pose (−10.4 kcal/mol) between entrectinib was obtained by structure-based docking (Figure 2G), showing the following results: the oxygen atom of amide group of entrectinib could interact with THR169 by forming 2 hydrogen bonds; 2 Nitrogen atoms of benzimidazole of entrectinib could bind together with the oxygen atom of THR317 by forming one hydrogen bond; the oxygen atom of tetrahydropyran of entrectinib interact with THR317 by forming a hydrogen bond; benzene of entrectinib binds with PHE168 by forming pi-pi stacking. Additionally, entrectinib’s structure participates in a hydrophobic interaction with ALA203, GLN320, PHE168, THR318, PRO316, THR352, VAL265, and THR352 of NDH-2 protein in *S. aureus* (Figure 2G).

### Disruption of the cell membrane by entrectinib against *Staphylococcus aureus*

**Figure 4:**
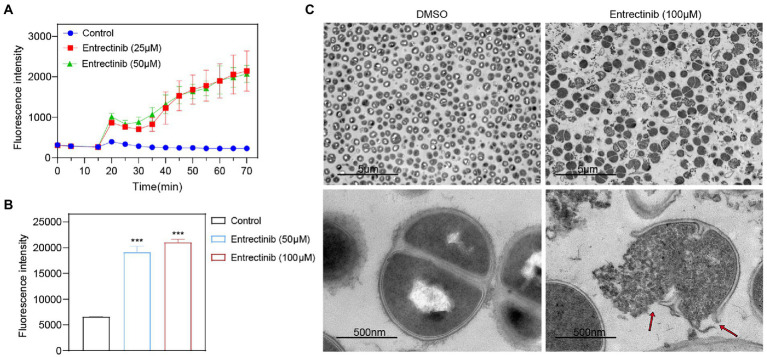
The influence of entrectinib on the cell membrane *Staphylococcus aureus*. **(A)** Effect of cytoplasmic membrane depolarization by entrectinib at 1× and 2× MIC concentrations. *S. aureus* cells were treated with DiBAC_4_(3) for 20 min and then treated with entrectinib for 50 min. The fluorescence of DiBAC_4_(3) was detected at an excitation at 492 nm with an emission at 518 nm. **(B)** Generation of ROS of *S. aureus* treated with entrectinib (2× and 4× MIC). *Staphylococcus aureus* cells are treated with DCFH-DA for 30 min, washed twice with PBS, then treated with entrectinib for 1 h and the fluorescence intensity was detected at an excitation wavelength of 488 nm and an emission wavelength of 525 nm. **(C)** The TEM characterization on bacteria cells treated with or without 100 μM entrectinib. Red arrows indicate disruption of cell membranes and loss of cell contents. Compared with control, ***p < 0.001; (Student’s *t*-test).

In order to determine the impact of entrectinib on the cell membrane of *S. aureus*, DiBAC4 (3) dye was used as an indicator of cytoplasmic membrane depolarization and our data exhibited the significant impact of entrectinib on the membrane depolarization of *S. aureus* (Figure 4A). To further confirm the damage of entrectinib on *S. aureus* cell membrane, transmission electron microscope (TEM) analysis was performed and our data demonstrated the cytoplasmic membrane damage and cytoplasmic content loss with entrectinib exposure (Figure 4C). Additionally, membrane permeability changes often result in the production of reactive oxygen species (ROS) and the overproduction of ROS involves in the destruction of proteins, DNA, membranes, and ultimately death of bacteria cell (Klinger-Strobel et al., 2016). In this study, the DCFH-DA fluorescent probe was used to detect ROS production in bacterial cells, showing that after entrectinib treatment for 1 h, the significantly increased ROS level of *S. aureus* when compared to that in the control group (Figure 4B).

## Discussion

Entrectinib is a potent multikinase inhibitor with systemic inhibition activity against multiple oncogenic kinases, such as neurotrophic tyrosine receptor kinase (NTRK), anaplastic lymphoma kinase (ALK) and proto-oncogene tyrosine protein kinase ROS1 (ROS1; Desai et al., 2022). Compared with similar drugs like larotrectinib and crizotinib, the designment of entrectinib facilitates it easily to penetrate the blood–brain barrier for the chemotherapy of tumors in the central nervous system (CNS; Doebele et al., 2020; Drilon et al., 2020). Entrectinib have been approved by FDA in 2019 and by Europe in 2020 clinically for the chemotherapy of solid tumors with positive NTRK fusions and non-small cell lung cancers with positive fusions of ROS1. This study firstly demonstrated that the MIC of entrectinib ranged from 25 to 50 μM, which showed a comparable high level compared with the commonly used anti-staphylococcus antibiotics, such as vancomycin and linezolid. Whereas the recommended dose of entrectinib used for adult patients is orally administered at 600 mg/day (Fischer et al., 2020; Frampton, 2021). Additionally, entrectinib is mainly metabolized in the liver primarily by cytochrome P450 isoenzymes CYP3A4 (the major route; Frampton, 2021). Therefore, the high dose of oral administration of entrectinib might facilitate its future use in the antimicrobial treatment of gastrointestinal infections. Moreover, combined with its good safety, entrectinib might be made as the paste for the local external application for the slight skin soft tissue infection or as fluid drops for the local eye infections. In local infectious tissue of chronic *S. aureus* infection, the biofilm formation might become a rational choice for eliminating the difficult-to-treat or biofilm-related *S. aureus*. Our data indicated the excellent anti-biofilm activity of entrectinib and further demonstrate its advantage in the future use of biofilm-related *S. aureus* infection.

In this study, the LC/MS-based proteomics data demonstrated the impact of entrectinib on the global expression of the proteins in *S. aureus*, including 12 significantly downregulated proteins with the functions closely correlated to structural constituent of the ribosome and structural molecular activity. Furthermore, the differentially expressed proteins with significantly increased levels were mainly correlated to the antioxidant activity, oxidoreductase activity, and protein-disulfide reductase activity. Worthy of our attention, the increased expression of Trx system-related proteins like TrxA, msrA2, and SAOUHSC_01999 were found (Lu and Holmgren, 2014). The thioredoxin system in *S. aureus* is composed of NADPH, thioredoxinreductase (TrxR), and thioredoxin (Trx; Holmgren, 1985; Dong et al., 2019). The electrons of several enzymes must be supplied by the thioredoxin system, which plays a crucial role in DNA synthesis, protein repair, and immune defense against oxidative stress in *S. aureus*. Additionally, some reports have indicated the antioxidant function of Trx system is involved in DNA and protein repair by reducing the expression level of the ribonucleotide reductase, methionine sulfoxide reductase, and regulating the activity of many redox-sensitive transcription factors (Gleason and Holmgren, 1988; Mostertz et al., 2008; Lu et al., 2013). Moreover, the Trx system provides electrons to thiol-dependent peroxidases (peroxiredoxins) for the rapid removal of reactive oxygen and nitrogen species. Previous studies support that TrxA in *S. aureus* defends against oxidative damage by donating electrons to mrsA and participating in protein repair (Potamitou et al., 2002; Lu and Holmgren, 2014). Overall, entrectinib inhibits *S. aureus* growth and biofilm formation possibly by inhibiting protein biosynthesis and producing reactive oxygen species.

Notably, entrectinib was able to penetrate and disrupt the integrity of bacterial cell membranes, suggesting that membrane targeting may play a critical role in the antimicrobial actions of entrectinib. The mechanism of action mediated the bactericidal effects of entrectinib was assessed by electron microscopy, further validating its initial damage to cell permeability and subsequently the cell membrane disruption of *S. aureus*. Whole-genome sequencing results showed that two SNPs were found in the gene encoding a membrane-bound dehydrogenase Type II NADH: quinoneoxidoreductase (NDH-2). NDH-2 is a member of the two-Dinucleotide Binding Domains Flavoprotein (tDBDF) superfamily and this superfamily has also been named as the flavin-disulfide reductases family. Previous reports have demonstrated the important roles of NDH-2involved in the respiratory chain andNDH-2 protein is the only NADH quinoneoxidoreductase expressed in *S. aureus* and absent in mammals (Sena et al., 2015; Marreiros et al., 2016; Blaza et al., 2017). Consequently, NDH-2is necessary for the *S. aureus* growth and has been regarded as a promising target for the development of antibacterial drugs. Furthermore, NDH-2 protein contributes to the maintenance of the NADH/NAD balance and participates in stabilizing the quinol/quinone ratio, which would avoid the excessive production of intracellular ROS (Melo et al., 2004; Marreiros et al., 2016; Sena et al., 2018). In this study, after entrectinib treatment, the increased ROS production in *S. aureus* might also contribute to NDH-2 gene mutations of the entrectinib-induced resistant clones. The molecular docking indicated the potential possible interaction of entrectinib with NDH-2 protein. Our data supported that NDH-2 protein was a possible binding target of entrectinib in this study, whereas their direct binding between entrectinib and NDH-2 need to be further confirmed. It is well-known that the bacterial cell membrane have been considered as a common target for developing the chemicals inhibiting bacterial growth and biofilm formations (Akhavan and Ghaderi, 2010; Yin et al., 2016). Our data robustly supported the disruption of entrectinib toward the cell membrane of *S. aureus*. Whereas, the mechanism and biological process of entrectinib disrupting the cell membrane of *S. aureus* should be elucidated in the future.

In addition, in this study, the *in vivo* experiments using a mouse wound infection model indicated the excellent antibacterial activity of entrectinib. Although the high dose of entrectinib with 600 mg/day was administered in patients with tumors, the pharmacodynamic characteristics of entrectinib should be further investigated. The drug concentration of intracellular entrectinib needs to be studied. Our data indicated the excellent anti-biofilm activities of entrectinib. And it might be feasible for entrectinib to be used for biofilm-related infection by clinically combining it with other commonly used antibiotics that have no effect on bacterial biofilm. Moreover, the synergetic antibacterial activity of entrectinib combined with other commonly used antibiotics, such as β-lactam, vancomycin, linezolid, and daptomycin, should be evaluated by *in vitro* and *in vivo* experiments. Furthermore, the ingredients of *S. aureus* cell membrane are complicated and the target details of the mechanical action of entrectinib on the cell membrane need to be investigated.

In conclusion, this study firstly demonstrated the antibacterial activity of entrectinib against *S. aureus* and could effectively inhibit the biofilm formation at sub-inhibitory concentrations. In addition, global proteomic analysis of entrectinib treated with *S. aureus* showed the involvement of the differentially increased expressed proteins in oxidoreductase activity, antioxidant activity, and protein-disulfide reductase activity. Entrectinib-induced resistant *S. aureus* clone can be selected by *in vitro* induction under entrectinib exposure and 3 amino acid mutations in the entrectinib-induced resistant strain, 2 of which were located in the gene encoding Type II NADH: quinoneoxidoreductase and one in GTP pyrophosphokinase family protein, were found. The interaction between entrectinib with NDH-2 was predicted by molecular docking. Finally, the bactericidal action of entrectinib on *S. aureus* might inhibit the bacterial growth by disrupting the cell membrane. Conclusively, entrectinib exhibit the antibacterial and anti-biofilm activity by disrupting the cell membrane.

## Data availability statement

The datasets presented in this study can be found in online repositories. The names of the repository/repositories and accession number(s) can be found at: The whole-genome sequencing files of the entrectinib-tolerant CHS101 clone were deposited in the NCBI database with the Biosample accession SAMN31720757 and the reference sequence the parenteral isolate CHS101 with the BioProject accession PRJNA889679. The raw data of whole-genome sequencing were posted in the Sequence Read Archive (SRA) database under BioProject accession number PRJNA901618 in NCBI. The raw proteomics data are deposited in the ProteomeXchange Consortium through the partner iProX system with dataset identifier PXD038342.

## Ethics statement

The animal study was reviewed and approved by The animal ethics Committee of 6th Affiliated Hospital of Shenzhen University Health Science Center.

## Author contributions

SL participated in the design of the study, carried out biofilm assay by crystal violet staining and cytotoxicity assay, predicted the binding model, analyzed whole-genome sequencing data and proteomics data, and drafted the manuscript. YX conducted the mice infection experiment and participated in whole-genome sequencing data analysis. HX participated in proteomics data analysis. ZW performed MIC and time-kill assays. JZ participated in biofilm assay. JZ and DL reviewed the manuscript. QD and ZY designed the study, participated in the data analysis, and provided critical revisions of the manuscript for valuable intellectual content. All authors contributed to the article and approved the submitted version.

## Funding

This work was supported by the National Natural Science Foundation of China (grant no. 82172283), Natural Science Foundation of Guangdong Province, China (grant nos. 2020A1515011049, 2020A1515010979, and 2021A1515011727), Shenzhen Key Medical Discipline Construction Fund (SZXK06162); Science, Technology, and Innovation Commission of Shenzhen Municipality of Key Funds and Basic Research Funds (grant nos. JCYJ 20180302144403714 and JCYJ20190809110209389), and the science funds of the Nanshan District Government (grant nos. NS2021117, NS2021140, NS2021144, and NS2021066).

## Conflict of interest

The authors declare that the research was conducted in the absence of any commercial or financial relationships that could be construed as a potential conflict of interest.

## Publisher’s note

All claims expressed in this article are solely those of the authors and do not necessarily represent those of their affiliated organizations, or those of the publisher, the editors and the reviewers. Any product that may be evaluated in this article, or claim that may be made by its manufacturer, is not guaranteed or endorsed by the publisher.
