# Supplementary material for: Inhibition of planktonic growth and biofilm formation of Staphylococcus aureus by entrectinib through disrupting the cell membrane
Source: Front Microbiol. 2023 Jan 9;13:1106319. doi: 10.3389/fmicb.2022.1106319 (PMC9868760; doi:10.3389/fmicb.2022.1106319)
Supplement: Supplementary file 1 [file Data_Sheet_1.PDF]

Table S1. Proteins expressed differently (up- or down-regulation) in *S. aureus* SA113 with 12.5 μM entrectinib exposure.

Uniprot ID	Log2FoldChange	Description
**Up-regulated**		
Q2G155	4.49	Lipase 2
Q2FWM8	4.42	Delta-hemolysin
Q2FWW1	3.17	MHC class II analog protein
Q2FXS4	2.66	DUF4930 family protein
Q2FXF9	2.64	Riboflavin biosynthesis protein RibD
Q2FXG2	2.6	6,7-dimethyl-8-ribityllumazine synthase
Q2FWR3	2.58	Phi ETA orf 22-like protein
Q2FV10	2.45	Betaine aldehyde dehydrogenase
Q2FVH0	2.39	Amino acid transporter, putative
Q2G2K8	2.21	Urease accessory protein UreE
Q2FZR7	2.2	ABC transmembrane type-1 domain-containing protein
Q2FX32	2.15	Conserved hypothetical phage protein
Q2FWN9	2.04	Uncharacterized leukocidin-like protein 2
Q2FV48	2	HTH tetR-type domain-containing protein
Q2G2G1	1.99	DoxX family protein
Q2FVK8	1.98	2,3-bisphosphoglycerate-dependent phosphoglycerate mutase
Q2FVI6	1.98	Viral A-type inclusion protein
Q2FUX3	1.96	Immunodominant staphylococcal antigen B
Q2FVK5	1.95	Immunoglobulin-binding protein Sbi
Q2FWQ9	1.89	Conserved hypothetical phage protein
Q2FWP0	1.88	Uncharacterized leukocidin-like protein 1
Q2FVG3	1.88	Carboxylic ester hydrolase
Q2FV63	1.85	Copper chaperone CopZ
Q2FXV3	1.85	Bac_luciferase domain-containing protein
Q2G1X0	1.81	Alpha-hemolysin
Q2FX56	1.74	Phage head protein, putative
Q2G2J2	1.74	Staphylococcal secretory antigen ssaA2
Q2FXJ6	1.72	Serine protease HtrA-like
Q2FYP0	1.72	Aspartate-semialdehyde dehydrogenase
Q2FVT5	1.72	Urocanate hydratase
Q2G1M1	1.72	Diacetyl reductase [(S)-acetoin forming]
Q2FZT8	1.71	Signal peptidase I
Q2FVB2	1.7	Fructose-1,6-bisphosphatase class 3
Q2G2P5	1.7	Nickel-binding protein NikA
Q2FXE2	1.66	Aldo_ket_red domain-containing protein
Q2G1I7	1.65	DUF4242 domain-containing protein
Q2FYU2	1.64	CAP domain-containing protein
Q2G1K8	1.61	Capsular polysaccharide biosynthesis protein, putative
Q2FX62	1.6	Phage structural protein, putative

P0A086	1.59	Peptide methionine sulfoxide reductase MsrA 2
Q2FYM9	1.58	Acylphosphatase
Q2G122	1.57	5-methyltetrahydropteroyltriglutamate--homocysteine methyltransferase
Q2FZR4	1.55	Oligopeptide ABC transporter, ATP-binding protein, putative
Q2G2D8	1.54	ABC transporter, substrate-binding protein, putative
Q2FYN7	1.54	2,3,4,5-tetrahydropyridine-2,6-dicarboxylate N-acetyltransferase
Q2FX91	1.54	Staphylococcal protein
Q2FZW4	1.51	D-alanyl carrier protein
Q2G2G0	1.5	DM13 domain-containing protein
Q2G093	1.49	Lipoteichoic acid synthase
Q2FZR5	1.47	ABC transporter domain-containing protein
Q2FX07	1.45	DUF2154 domain-containing protein
Q2FYK2	1.44	Nfu_N domain-containing protein
Q2G000	1.43	Thioredoxin, putative
Q2FZD2	1.4	Thioredoxin
Q2FVD5	1.4	Uncharacterized oxidoreductase
Q2FX34	1.38	Single-strand DNA-binding protein, putative
Q2G087	1.38	Histidinol-phosphate aminotransferase
Q2FVV8	1.36	Transcriptional regulator, putative
Q2G1J0	1.36	Putative aldehyde dehydrogenase AldA
Q2FX08	1.36	Sensor protein VraS
Q2G2E0	1.33	Na_H_Exchanger domain-containing protein
Q2FZK7	1.28	Bifunctional autolysin
Q2FX13	1.28	UPF0435 protein SAOUHSC_02093
Q2G0K4	1.26	Proline/betaine transporter, putative
Q2FYP1	1.26	Aspartokinase
Q2G2S6	1.25	Foldase protein PrsA
Q9EZ12	1.25	4-hydroxy-tetrahydrodipicolinate synthase
Q2FWZ8	1.25	Bacterial non-heme ferritin
Q2FXL6	1.24	Putative universal stress protein SAOUHSC_01819
Q2FXK1	1.24	D-3-phosphoglycerate dehydrogenase
Q2FVG8	1.23	Amino acid ABC transporter, ATP-binding protein, putative
Q9EZ08	1.22	Alanine racemase 2
Q2G2U8	1.22	Putative antiporter subunit mnhA2
Q2G280	1.21	NADH-dependent peroxiredoxin
Q2G2U3	1.21	YycH domain-containing protein
Q2FXI6	1.2	Thioredoxin domain-containing protein
Q2G2D5	1.19	6-phospho-beta-galactosidase
Q2G0E2	1.19	N-acetyltransferase domain-containing protein
Q2FYN6	1.17	Uncharacterized hydrolase SAOUHSC_01399
Q2G1I3	1.17	Isovaleryl-CoA dehydrogenase
Q2FYU7	1.15	Catalase
Q2FX09	1.14	Response regulator protein VraR
Q2G2C9	1.14	Ribosomal_L7Ae domain-containing protein

Q2FZU7	1.13	FMN oxidoreductase, putative
Q93Q23	1.12	Monofunctional glycosyltransferase
Q2G015	1.12	Clumping factor A
Q2FZT0	1.11	FeS_assembly_P domain-containing protein
Q2G2N7	1.11	Phage terminase, small subunit, putative
Q2FYF1	1.1	Elastin-binding protein EbpS
Q2G1F1	1.1	Lysostaphin
Q2FZJ1	1.09	Phosphoribosylformylglycinamidine synthase subunit PurQ
P52078	1.08	Uncharacterized protein SAOUHSC_00997
Q9EZ11	1.08	4-hydroxy-tetrahydrodipicolinate reductase
Q2FZY9	1.08	UPF0337 protein SAOUHSC_00845
Q2FX55	1.07	Conserved hypothetical phage protein
Q2G150	1.06	Lipoyl-binding domain-containing protein
Q2G0G1	1.04	Alcohol dehydrogenase
Q2FYL3	1.04	Probable CtpA-like serine protease
Q2FXG1	1.03	Riboflavin biosynthesis protein RibBA
Q2FWX9	1.02	4,4'-diaponeurosporen-aldehyde dehydrogenase
Q2FYS7	1.02	Uncharacterized protein SAOUHSC_01349
Q2FVA6	1.02	N-acetyltransferase
Q2G1J2	1.02	Heme oxygenase (staphylobilin-producing) 2
Q2FYE8	1.02	Ferredoxin, putative
Q2FUW9	1.01	Surface protein F
Q2G0G3	1	HD domain-containing protein
**Down-regulated**		
Q2FVM1	-2.97	Nitrate reductase (quinone)
Q2FWX4	-2.85	DUF4097 domain-containing protein
Q2FYJ3	-2.55	L-threonine dehydratase catabolic TdcB
Q2FYJ2	-2.44	Alanine dehydrogenase 1
Q2FXW2	-2.39	UPF0473 protein SAOUHSC_01719
Q2G1X6	-2.26	7-cyano-7-deazaguanine synthase
Q2FZ73	-1.95	Carbamoyl-phosphate synthase small chain
Q2G079	-1.86	Protein NrdI
Q2FW14	-1.83	50S ribosomal protein L29
Q2FVM7	-1.83	Oxygen regulatory protein NreC
Q2FVB4	-1.69	ABC transporter domain-containing protein
Q2FXP3	-1.67	Transcriptional repressor NrdR
Q2FX15	-1.66	HotDog ACOT-type domain-containing protein
Q2FXX9	-1.66	YqeG family HAD IIIA-type phosphatase
Q2FVL8	-1.65	Assimilatory nitrite reductase [NAD(P)H], large subunit, putative
P0A0G2	-1.64	50S ribosomal protein L30
Q2G2V4	-1.64	Putative membrane protein insertion efficiency factor
Q2FXW5	-1.52	Peptidase_U32_C domain-containing protein

Q2FV28	-1.52	Epoxyqueuosine reductase QueH
Q9RFJ6	-1.52	HTH-type transcriptional regulator rot
Q2G1E3	-1.49	Isoprenylcysteine carboxyl methyltransferase
Q2FW17	-1.46	50S ribosomal protein L24
Q2FUS7	-1.46	tRNA uridine(34) hydroxylase
Q2FZM8	-1.42	Lipoprotein
Q2G188	-1.4	Type VII secretion system accessory factor EsaA
Q2G090	-1.4	DNA helicase
Q2FVH3	-1.39	2-dehydropantoate 2-reductase
Q2FZM6	-1.39	Glycosyl transferase, group 1
Q2G2Q3	-1.39	tRNA pseudouridine synthase B
Q2FV53	-1.37	N-acetyltransferase domain-containing protein
Q2FZ72	-1.36	Carbamoyl-phosphate synthase large chain
Q2FYY8	-1.36	Aluminum resistance protein
Q2FZP4	-1.36	Peptide chain release factor 3
Q2FW29	-1.34	50S ribosomal protein L36
Q2FXY6	-1.33	30S ribosomal protein S20
Q2FZU1	-1.33	Argininosuccinate synthase
Q2FWL5	-1.31	ABC transporter, ATP-binding protein, putative
Q2G2Q8	-1.31	TIGR01741 family protein
Q2FXT0	-1.3	50S ribosomal protein L27
Q2FYK0	-1.27	Probable queuosine precursor transporter
Q2FWC7	-1.23	Type II pantothenate kinase
Q2FZ74	-1.22	Dihydroorotase
Q2G0B8	-1.22	N-acetyltransferase domain-containing protein
Q2FYP2	-1.21	ABC transporter, ATP-binding protein, putative
Q2FX98	-1.2	HTH cro/C1-type domain-containing protein
Q2FVL9	-1.2	Assimilatory nitrite reductase [NAD(P)H], small subunit, putative
Q2FXI2	-1.19	tRNA (guanine-N(7)-)-methyltransferase
Q2G189	-1.18	Type VII secretion system extracellular protein A
Q2FVN1	-1.17	Probable nitrate transporter NarT
Q2FVS7	-1.16	Aldose 1-epimerase
P0A0H0	-1.15	30S ribosomal protein S12
Q2FW78	-1.13	Membrane protein, putative
Q2FYK3	-1.12	Conserved virulence factor C
Q2G2W1	-1.09	Drug resistance transporter, EmrB/QacA subfamily, putative
Q2FZ42	-1.08	50S ribosomal protein L19
Q2FW10	-1.08	30S ribosomal protein S19
Q2FXY5	-1.08	DNA_pol3_delta domain-containing protein
Q2FZ45	-1.06	30S ribosomal protein S16
Q2FZW0	-1.05	Pyr_redox_2 domain-containing protein
Q9RQP7	-1.05	Poly-beta-1,6-N-acetyl-D-glucosamine N-deacetylase
Q2FYT9	-1.03	UPF0291 protein SAOUHSC_01336
Q2G0B5	-1.02	DUF1014-domain-containing protein

Q2FXM2	-1.02	Cytosolic protein containing multiple CBS domains
Q2FXZ4	-1.01	Ribosomal protein L11 methyltransferase
Q2FW12	-1	30S ribosomal protein S3
Q2G0N7	-1	MTS domain-containing protein
Q2FX16	-1	Conserved hypothetical phage protein

Table S2. Mutations in the entrectinib-induced non-sensitive isolate detected by the whole-genome sequencing

**Ref_gene_ID**	**NA mutations**	**AA mutations**	**Subject description**
CHS101_GM000493	T412A	S138T	Type II NADH:quinone oxidoreductase
CHS101_GM000493	C413A	T138K	Type II NADH:quinone oxidoreductase
CHS101_GM000426	G586A	A196T	GTP pyrophosphokinase family protein
